# Weyl Fermions in VI_3_ Monolayer

**DOI:** 10.3389/fchem.2020.00722

**Published:** 2020-08-26

**Authors:** Taoyuan Jia, Weizhen Meng, Haopeng Zhang, Chunhai Liu, Xuefang Dai, Xiaoming Zhang, Guodong Liu

**Affiliations:** School of Material Sciences and Engineering, Hebei University of Technology, Tianjin, China

**Keywords:** topological semimetal, 2D materials, first-principles calculations, half-metal, Weyl state

## Abstract

We report the presence of a Weyl fermion in VI_3_ monolayer. The material shows a sandwich-like hexagonal structure and stable phonon spectrum. It has a half-metal band structure, where only the bands in one spin channel cross the Fermi level. There are three pairs of Weyl points slightly below the Fermi level in spin-up channel. The Weyl points show a clean band structure and are characterized by clear Fermi arcs edge state. The effects of spin-orbit coupling, electron correlation, and lattice strain on the electronic band structure were investigated. We find that the half-metallicity and Weyl points are robust against these perturbations. Our work suggests VI_3_ monolayer is an excellent Weyl half-metal.

## Introduction

In recent years, Weyl semimetals (WSMs) have attracted extensive research attentions (Wan et al., 2011; Lv et al., 2015; Shekhar et al., 2015; Soluyanov et al., 2015; Sun et al., 2015; Weng et al., 2015; Deng et al., 2016; Koepernik et al., 2016; Wu et al., 2016; Kumar et al., 2017). In a WSM, at least one of the time reversal and inversion symmetry is broken. The crossing points, namely, Weyl nodes, appear in pairs with different chirality (Ruan et al., 2016a,b). Such chiral anomaly can induce interesting transport properties such as anomalous Hall effect and negative magnetoresistance (Liu et al., 2013; Son and Spivak, 2013; Liu and Vanderbilt, 2014; Hirayama et al., 2015; Huang et al., 2015). Besides, Weyl nodes can be classified into two categories, namely, types I and II, according to the tilt degree of band crossing. Type I WSMs with traditional band dispersion follow the Lorentz symmetry (Wan et al., 2011; Lv et al., 2015; Shekhar et al., 2015; Sun et al., 2015; Weng et al., 2015). However, for type II WSMs (Soluyanov et al., 2015; Deng et al., 2016; Koepernik et al., 2016; Wu et al., 2016; Kumar et al., 2017), the Weyl cones are completely tilted. The tilted Weyl cones can cause the coexistence of electron-like pocket and hole-like pocket at the same energy level. As the results, type II WSMs have different physical phenomena from type I ones, including modified anomalous Hall conductivity, direction-dependent chiral anomaly, and momentum space Klein tunneling (Koshino, 2016; O'Brien et al., 2016; Yu et al., 2016; Zyuzin and Tiwari, 2016).

Up to now, a large number of WSMs have been reported, and some of which have been confirmed in experiments such as TaAs (Lv et al., 2015), NbP (Shekhar et al., 2015), and NbAs (Yang et al., 2019). These examples are all three-dimensional (3D) non-magnetic materials. Recently, WSMs in two-dimensional (2D) materials and magnetic materials have received increasing interests. For 2D WSMs, the interest arises from the promising applications in spintronic nanodevices (You et al., 2019). For magnetic WSMs, the interest comes from the novel interplay between the non-trivial band topology and the magnetic ordering (Xu et al., 2011; Kübler and Felser, 2016; Wang et al., 2016; Chen et al., 2019; He et al., 2019; Jin et al., 2020; Meng et al., 2020a,b,c). Recently, the VI_3_ material (Kong et al., 2019; Tian et al., 2019; Huang et al., 2020; Long et al., 2020; Zhang et al., 2020), both in 3D and 2D, has attracted great attention. The 3D VI_3_ material is a ferromagnetic insulator, which is a layered material (Kong et al., 2019). In addition, 3D VI_3_ compound has a R3 phase at room temperature, and experimental evidence suggests that it may undergo a structure phase transition at 78 K (Huang et al., 2020). Importantly, 2D VI_3_ had been proved by Long et al. (2020) to be a ferromagnetic half-metal and provides an excellent candidate material for electronic devices. In this work, we report that VI_3_ monolayer is an excellent 2D WSM. We have systematically investigated the stability, magnetism, and band topology of VI_3_ monolayer. We find VI_3_ monolayer is dynamically stable and naturally has the ferromagnetic ordering. The band structure suggests VI_3_ monolayer is a half-metal, which holds fully spin-polarized conducting electrons. Especially, there exists a band crossing near the Fermi level, which forms three pairs of Weyl fermions in the spin-up band structure. We have further investigated the effects of spin-orbit coupling (SOC), electron correlation, and lattice strain on the electronic band structure. In addition, the non-trivial edge states for the Weyl points are clearly identified. These results suggest VI_3_ monolayer can serve as a good platform to investigate Weyl states in 2D.

## Computational Methods and Details

The first-principles calculations in this work are performed by using the Vienna *ab initio* Simulation Package (Blochl, 1994; Kresse and Joubert, 1999). The exchange-correlation potential is adopted by the generalized gradient approximation (GGA) of Perdew–Burke–Ernzerhof functional (Perdew et al., 1996). For the crystal structure of VI_3_ monolayer, we built a vacuum with the thickness >18 Å to avoid potential interactions between layers. The cutoff energy is set as 500 eV. The Brillouin zone is sampled by a Monkhorst–Pack k-mesh with size of 15 × 15 × 1. To account for the Coulomb interaction, the GGA + U method is applied during our calculations (Anisimov et al., 1991). For the V-3d orbitals, the *U* value is chosen as 3eV. The phonon spectra are calculated by using the PHONOPY code (Togo et al., 2008).

## Results and Discussion

Before studying the structure and electronic band of the monolayer VI_3_, we want to point out that the layered compound VI_3_ has already been synthesized by chemical vapor transport method experimentally. The specific synthesis process can be found in Kong et al. (2019). In addition, monolayer VI_3_ is very promising to be exfoliated from its bulk phase (Miro et al., 2014).

**Figure 1:**
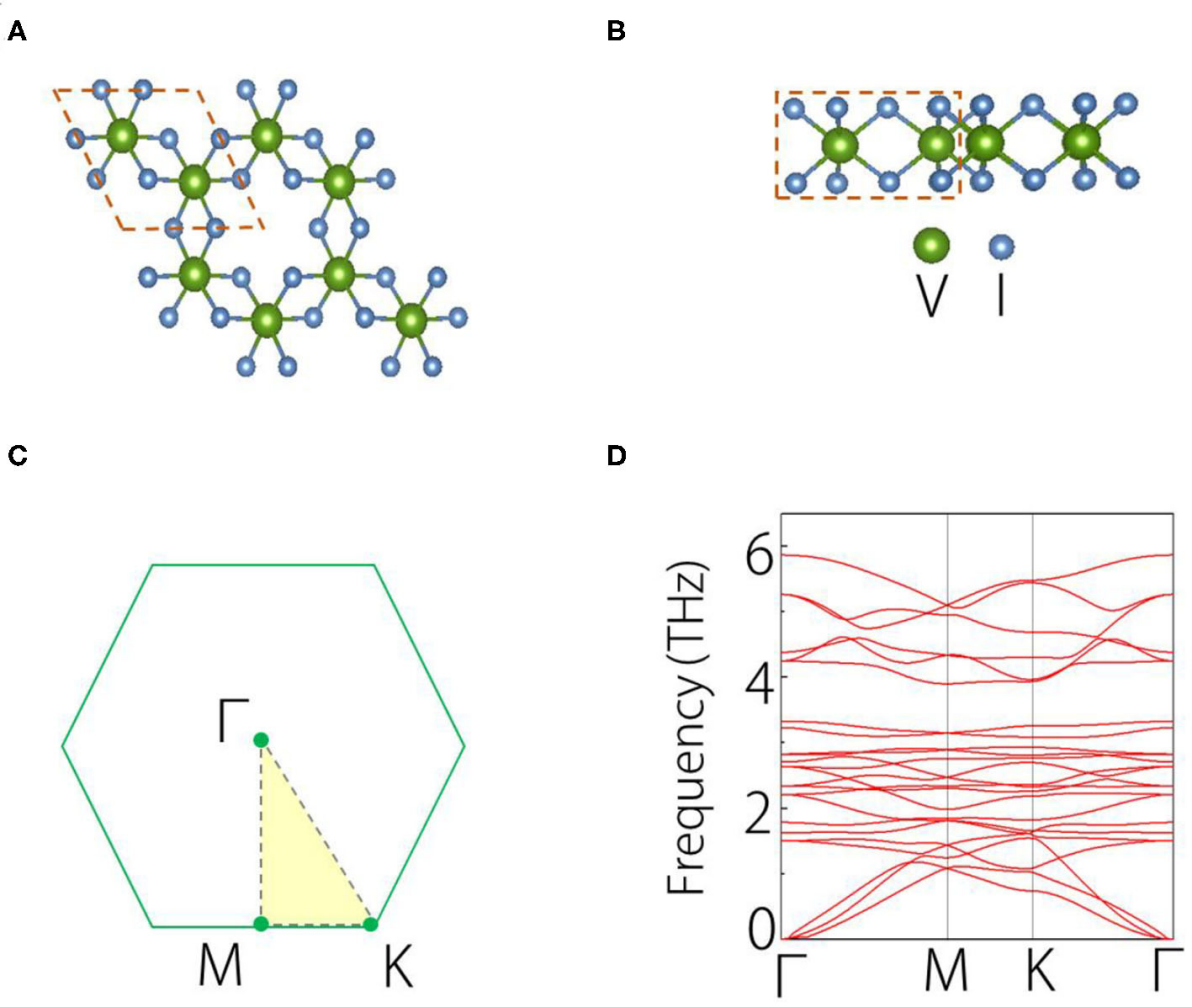
**(A)** Top view and **(B)** side views of VI_3_ monolayer, the dotted line circles the primitive cell. **(C)** Brillouin zone for VI_3_ monolayer. **(D)** Calculated phonon spectrum of VI_3_ monolayer.

Next, we study the structure of single-layer VI_3_. Figures 1A,B show the top and side views of the geometric structure of VI_3_ monolayer. Figure 1C shows the Brillouin zone (BZ) of monolayer VI3. In the structure, each V atom bonds with six I atoms, forming the hexagonal structure. From the side view, we can observe that the material has a triple-layered form, with one V layer sandwiched by two I layers. The circled regions in (A) and (B) show the unit cell of VI_3_ monolayer, which contains two V atoms and six I atoms. The Cr_2_C compound shows a hexagonal structure with space group *P3M1*. The lattice constant of VI_3_ monolayer is *a* = *b* = 7.13 Å. The bond length of V-I is 2.80 Å, and that of I-I is 4.13 Å. These values are very close to the reported results of He et al. (2016). We have calculated the phonon spectrum of VI_3_ monolayer, as displayed in Figure 1D. We find no negative frequency phonons in all the highly symmetric *k* paths. This suggests that the VI_3_ monolayer can be stable.

Before studying the band structure of VI_3_ monolayer, we first verify its magnetic ground state. In VI_3_ monolayer, the magnetic moments are mainly contributed by the 3d transition element V. Here, we consider three potential magnetization directions of V moment, including [001], [010], and [100]. Our calculation results show that the [001] magnetization direction has the lowest energy. Then, in the [001] direction, we considered four magnetic configurations including ferromagnet (FM), Néel antiferromagnet (AFM), stripe AFM, and zigzag AFM. Our calculation results show that the energy of FM is lower than that of other magnetic structures in VI_3_ monolayer. The total magnetic moment is 4 μ_B_ per unit cell, which is almost contributed by the V atoms. In addition, we have calculated the exchange energy Δ*E* of VI_3_ monolayer, which is approximately 28 meV. Then, we can estimate the Curie temperature (*T*_*c*_) according to the following equation:

(1) $$\begin{array}{l} k_{\text{B}}T_{\text{c}}=2\Delta /\left(3C\right) \end{array}$$

In (1), the parameter “*C*” represents the number of magnetic atoms in unit cell, and “*k*_B_” represents the Boltzmann constant. We calculated that the Curie temperature *T*_c_ value of VI_3_ monolayer is 106 K, which is comparable with the Monte Carlo simulations (98 K) (He et al., 2016).

**Figure 2:**
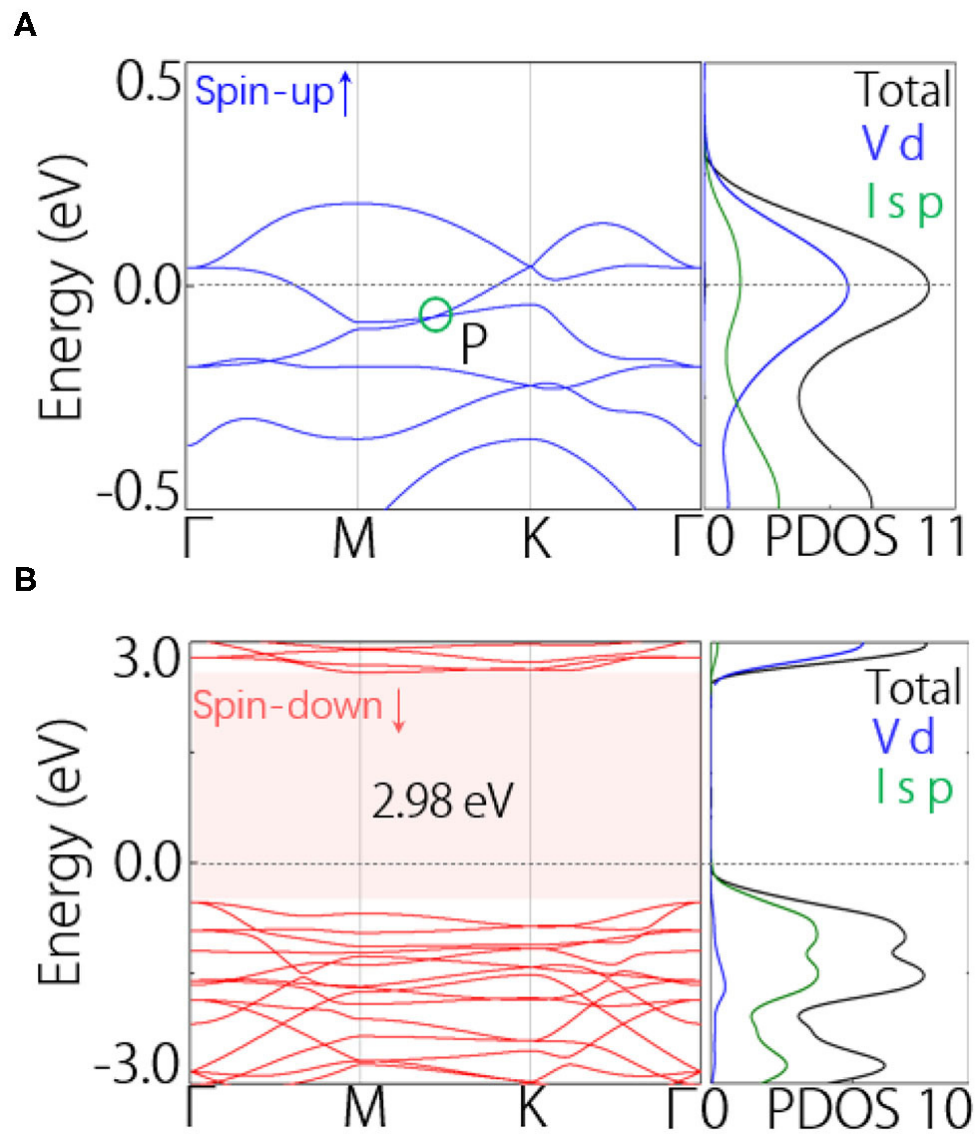
**(A)** Spin-up and **(B)** spin-down band structures of VI_3_ monolayer without SOC. In **(A,B)**, the total and projected density of states (PDOS) are also shown.

Here, we discuss the electronic band structure of VI_3_ monolayer. At first, we did not consider SOC in the calculations. The band structures are shown in Figures 2A,B. In the spin-up band structure, we find one band crosses the Fermi level, manifesting a metallic signature (Figure 2A). In the spin-down band structure, we can find a band gap around the Fermi level, manifesting the insulating signature (Figure 2B). Therefore, VI_3_ monolayer is in fact a half-metal. In particular, the half-metal gap is about 2.98 eV (Figure 2B), which is much layer than previously reported half-metals in 2D including YN_2_ (1.35eV) (Liu et al., 2017) and Na_2_C (0.77eV) monolayer (Ji et al., 2018). In the spin-up band structure, we notice there shows a band crossing point (P) in the M–K path, slightly below the Fermi level (Figure 2B). By checking the density of states, we find the states near the Fermi level mostly come from the *d* orbitals of V atom.

**Figure 3:**
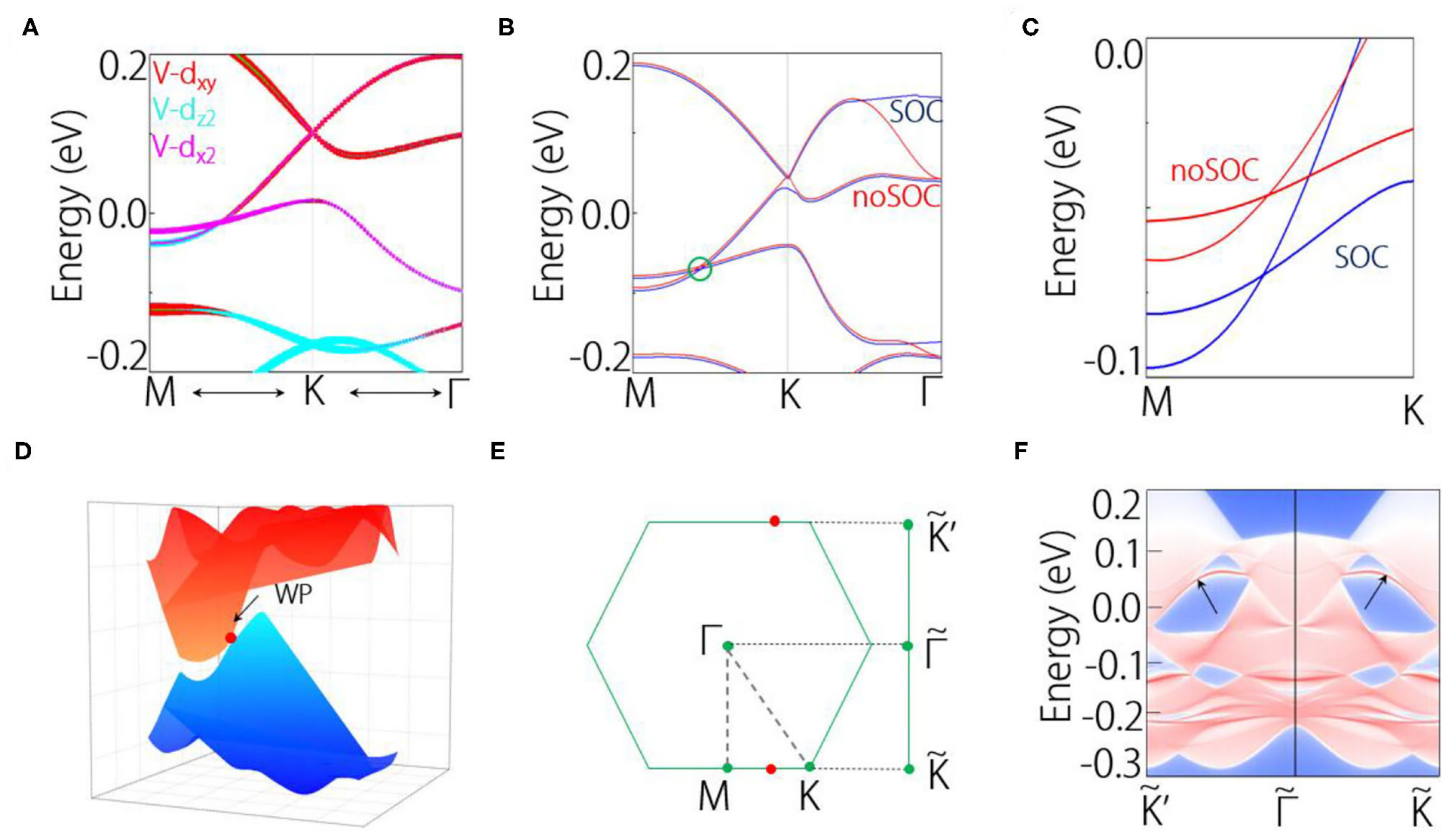
**(A)** Orbital-projected band structure near the crossing point P. **(B,C)** Comparison of band structure between SOC and without SOC, where **(C)** is the enlarged band structure. **(D)** 3D band dispersions near the Weyl point. **(E)** 2D Brillouin zone and its projection to the edge. **(F)** Edge states of VI_3_ monolayer.

Figure 3A shows the orbital-projected band structure near the crossing point P. We can observe the band inversion of the two bands, suggesting the non-trivial band topology in VI_3_ monolayer. Then we take into account the SOC effect in the calculation. The comparison of band structure between SOC and without SOC is shown in Figures 3B,C. We can find that the band crossing retains under SOC, even though its position has slightly changed. Without SOC, the crossing point locates at 0.04 eV below the Fermi level; under SOC, it locates at 0.08 eV below the Fermi level. Because the band crossing happens in two bands, the band crossing in fact forms Weyl points. To be noted, in VI_3_ monolayer, the time reversal is broken, but the inversion symmetry is retained; hence, the Weyl points are time-reversal–breaking Weyl points.

In Figure 3D, we show the 3D plotting of band structure near the Weyl point. We can find that the Weyl cone is titled. Because of the preserved inversion symmetry, there are in total three pairs of such Weyl points in the system. From the symmetry analysis, the Weyl points in monolayer VI_3_ are protected by the C_3v_ symmetry. It is worth noticing that Weyl points have many special physical phenomena, such as modified anomalous Hall conductivity, direction-dependent chiral anomaly, momentum space Klein tunneling (Koshino, 2016; O'Brien et al., 2016; Yu et al., 2016; Zyuzin and Tiwari, 2016). However, the Weyl fermions have been rarely found in 2D materials. Therefore, the VI_3_ monolayer reported here can be a good platform to study the Weyl fermions in 2D. In addition, in Figure 3E, the orbital projection is performed on (010) surface, we have also identified the Fermi arc edge states of the Weyl points, as shown in Figure 3F. This further verifies the non-trivial band topology in VI_3_ monolayer.

**Figure 4:**
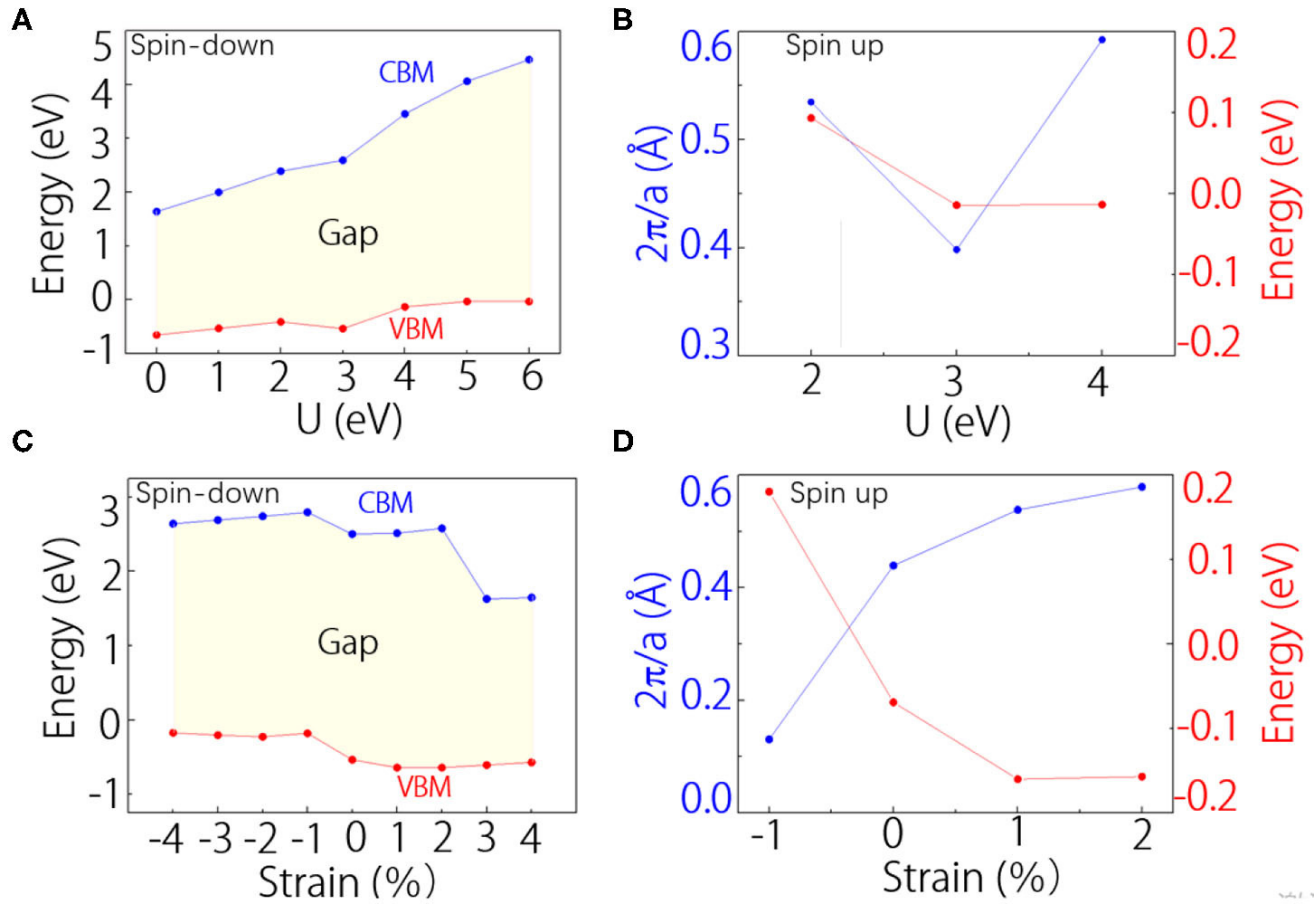
**(A)** The curves of the valence band maximum (VBM) and the conduction band minimum (CBM) under different *U*-values in the spin-down channel. The shadowed area shows the band gap. **(B)** The position of Weyl points under different *U*-values. **(C,D)** Similar with **(A,B)**, but for the changes under different stains.

Finally, we discuss the effects of electron correlation and lattice strain on the half-metal band structure and the Weyl points. In Figure 4A, we show the positions of the conduction band minimum and the valence band maximum with shifting the *U*-values of V atom from 0 to 6 eV. The results show that VI_3_ monolayer is always a ferromagnetic half-metal, and the spin-down band gap will increase with increasing the *U*-values. For the Weyl points, we find they can exist when *U*-values are at 2–4 eV. Figure 4B shows the positions of the Weyl points at different *U*-values. Similarly, in Figures 4C,D, we show the strain effects on the half-metal band structure and the Weyl points. We can find that the half-metal band structure can retain from 4% compressive stain to 4% tensile strain (Figure 4C). Meanwhile, as shown in Figure 4D, we find the Weyl points can exist from 1% compressive stain to 2% tensile strain (Figure 4D). These results show that the half-metal band structure and the Weyl points are in some degree robust against electron correlation effects and lattice strain.

## Summary

We have reported the Weyl fermion in 2D VI_3_ monolayer. The phonon spectrum suggests VI_3_ monolayer is dynamically stable. We have verified that VI_3_ monolayer has the ferromagnetic ground state. In the ground state, we find VI_3_ monolayer has a half-metal band structure with the half-metallic gap as large as 2.98 eV; thus, the conducting electrons can be fully spin-polarized. Very interestingly, VI_3_ monolayer shows three pairs of Weyl points near the Fermi level, locating in the spin-up band structure. Importantly, the three pairs of Weyl points show clear Fermi arcs on the edge. Moreover, we verify that the half-metal band structure and the Weyl points in VI_3_ monolayer are robust against proper electron correlation effects and lattice strain. These properties make VI_3_ monolayer have promising applications in spintronic nanodevices.

## Data Availability Statement

The raw data supporting the conclusions of this article will be made available by the authors, without undue reservation.

## Author Contributions

This project was conceived by XZ and GL. TJ, HZ, and CL completed theoretical model and first principles calculations with the help from XZ and XD. TJ and WM performed the data analysis and designed the manuscript with the assistances from XZ and GL. This manuscript was written by TJ and XZ with important inputs from GL and XD. This project was supervised by GL. All authors discussed the results and commented on the manuscript.

## Conflict of Interest

The authors declare that the research was conducted in the absence of any commercial or financial relationships that could be construed as a potential conflict of interest.

## References

[B1] Anisimov V. I. Zaanen J. Andersen O. K. (1991). Band theory and mott insulators: Hubbard U instead of stoner I. Phys. Rev. B 44:943. 10.1103/PhysRevB.44.943 9999600

[B2] Blochl P. E. (1994). Projector augmented-wave method. Phys. Rev. B 50:17953. 10.1103/PhysRevB.50.17953 9976227

[B3] Chen C. Yu Z. M. Li S. Chen Z. Sheng X. L. Yang S. A. (2019). Weyl-loop half-metal in Li_2_ (FeO_3_) _2_. Phys. Rev. B 99:075131 10.1103/PhysRevB.99.075131

[B4] Deng K. Wan G. L. Deng P. Zhang K. N. Ding E. Y. Yan M. Z. (2016). Experimental observation of topological Fermi arcs in type-II Weyl semimetal MoTe_2_. Nat. Phys. 12, 1105–1110. 10.1038/nphys3871

[B5] He J. J. Ma S. Y. Lyu P. Nachtigall P. (2016). Unusual Dirac half-metallicity with intrinsic ferromagnetism in vanadium trihalide monolayers. J. Mater. Chem. C 4, 2518–2526. 10.1039/C6TC00409A

[B6] He T. L. Zhang X. M. Meng W. Z. Jin L. Dai X. F. Liu G. D. (2019). Topological nodal lines and nodal points in the antiferromagnetic material β-Fe_2_PO_5_. J. Mater. Chem. C 7, 12657–12663. 10.1039/C9TC04046C

[B7] Hirayama M. Okugawa R. Ishibashi S. Murakami S. Miyake T. (2015). Weyl node and spin texture in trigonal tellurium and selenium. Phys. Rev. Lett. 114:206401. 10.1103/PhysRevLett.114.206401 26047243

[B8] Huang C. X. Wu F. Yu S. L. Jena P. Kan E. (2020). Discovery of twin orbital-order phases in ferromagnetic semiconducting VI3 monolayer. Phys. Chem. Phys. 22, 512–517. 10.1039/C9CP05643B 31828254

[B9] Huang X. Zhao L. Long Y. Wang P. Chen D. Yang Z. (2015). Observation of the chiral-anomaly-induced negative magnetoresistance in 3D Weyl semimetal TaAs. Phys. Rev. X 5:031023 10.1103/PhysRevX.5.031023

[B10] Ji W. X. Zhang B. M. Zhang S. F. Zhang C. W. Ding M. Wang P.. (2018). Na_2_C monolayer: a novel 2*p* Dirac half-metal with multiple symmetry-protected Dirac cones. Nanoscale 10, 13645–13651. 10.1039/C8NR02761G 29985502

[B11] Jin L. Zhang X. M. He T. L. Meng W. Z. Dai X. F. Liu G. D. (2020). Ferromagnetic two-dimensional metal-chlorides MCl (M= Sc, Y, and La): candidates for Weyl nodal line semimetals with small spin-orbit coupling gaps. App. Sur. Sci. 520:146376 10.1016/j.apsusc.2020.146376

[B12] Koepernik K. Kasinathan D. Efremov D. V. Khim S. Borisenko S. Buchner B. (2016). TaIrTe_4_: a ternary type-II Weyl semimetal. Phys. Rev. B 93:201101 10.1103/PhysRevB.93.201101

[B13] Kong T. Stolze K. Timmons E. I. Tao J. Ni D. Guo S.. (2019). VI3-a new layered ferromagnetic semiconductor. Adv Mater. 31:1808074. 10.1002/adma.201808074 30843286

[B14] Koshino M. (2016). Cyclotron resonance of figure-of-eight orbits in a type-II Weyl semimetal. Phys. Rev. B 94:035202 10.1103/PhysRevB.94.035202

[B15] Kresse G. Joubert D. (1999). From ultrasoft pseudopotentials to the projector augmentedwave method. Phys. Rev. B 59:1758 10.1103/PhysRevB.59.1758

[B16] Kübler J. Felser C. (2016). Non-collinear antiferromagnets and the anomalous Hall effect. Europhys. Lett. 114:47005 10.1209/0295-5075/114/47005

[B17] Kumar N. Sun Y. Xu N. Manna K. Yao M. Y. Suss V.. (2017). Extremely high magnetoresistance and conductivity in the type-II Weyl semimetals WP_2_ and MoP_2_. Nat. Commun. 8:1642. 10.1038/s41467-017-01758-z 29158479 PMC5696372

[B18] Liu C. X. Ye P. Qi X. L. (2013). Chiral gauge field and axial anomaly in a Weyl semimetal. Phys. Rev. B 87:235306 10.1103/PhysRevB.87.235306

[B19] Liu Z. F. Liu J. Y. Zhao J. J. (2017). YN_2_ monolayer: novel p-state Dirac half metal for high-speed spintronics. Nano Res. 10, 1972–1979. 10.1007/s12274-016-1384-3

[B20] Liu J Vanderbilt D. (2014). Weyl semimetals from noncentrosymmetric topological insulators. Phys. Rev. B 90:155316. 10.1103/PhysRevB.90.15531627935725

[B21] Long C. Wang T. Jin H. Wang H. Dai Y. (2020). Stacking-independent ferromagnetism in Bilayer VI3 with Half-metallic characteristic. J. Phys. Chem. Lett. 11, 2158–2164. 10.1021/acs.jpclett.0c00065 32105479

[B22] Lv B. Q. Weng H. M. Fu B. B. Wang X. P. Miao H. Ma J. (2015). Experimental discovery of Weyl semimetal TaAs. Phys. Rev. X 5:031013 10.1103/PhysRevX.5.031013

[B23] Meng W. Z. Zhang X. M. He T. L. Dai X. F. Jin L. Liu G. D. (2020b). Crystal Structures, electronic structures and topological signatures in equiatomic TT'X compounds (T = Sc, Zr, Hf; T' = Co, Pt, Pd, Ir, Rh; X = Al, Ga, Sn). J. Phy. Chem. C 124, 7378–7385. 10.1021/acs.jpcc.0c00303

[B24] Meng W. Z. Zhang X. M. He T. L. Dai X. F. Jin L. Liu Y.. (2020a). Ternary compound HfCuP: An excellent Weyl semimetal with the coexistence of type-I and type-II Weyl nodes. J. Adv. Res. 24, 523–528. 10.1016/j.jare.2020.05.026 32612858 PMC7320317

[B25] Meng W. Z. Zhang X. M. Liu Y. Dai X. F. Liu G. D. (2020c). Lorentz-violating type-II Dirac fermions in full-Heusler compounds XMg2Ag (X=Pr, Nd, Sm). New J. Phys. 22:073061 10.1088/1367-2630/ab9d55

[B26] Miro P. Audiffred M. Heine T. (2014). An atlas of two-dimensional materials. Chem. Soc. Rev. 43, 6537–6554. 10.1039/C4CS00102H 24825454

[B27] O'Brien T. E. Diez M. Beenakker C. W. J. (2016). Magnetic breakdown and Klein tunneling in a type-II Weyl semimetal. Phys. Rev. Lett. 116:236401. 10.1103/PhysRevLett.116.236401 27341246

[B28] Perdew J. P. Burke K. Ernzerhof M. (1996). Generalized gradient approximation made simple. Phys. Rev. Lett. 77:3865. 10.1103/PhysRevLett.77.3865 10062328

[B29] Ruan J. Jian S. K. Yao H. Zhang H. Zhang S. C. Xing D. (2016a). Symmetry-protected ideal 14 Weyl semimetal in HgTe-class materials. Nat. Commun. 7:11136. 10.1038/ncomms11136 27033588 PMC4822222

[B30] Ruan J. Jian S. K. Zhang D. Yao H. Zhang H. Zhang S. C.. (2016b). Ideal Weyl Semimetals in the Chalcopyrites CuTlSe_2_, AgTlTe_2_, AuTlTe_2_, and ZnPbAs2. Phys. Rev. Lett. 116:226801. 10.1103/PhysRevLett.116.226801 27314733

[B31] Shekhar C. Nayak A. K. Sun Y. Schmidt M. Nicklas M. Leermakers I. (2015). Extremely large magnetoresistance and ultrahigh mobility in the topological Weyl semimetal candidate NbP. Nat. Phys. 11, 645–649. 10.1038/nphys3372

[B32] Soluyanov A. A. Gresch D. Wang Z. J. Wu Q. S. Troyer M. Dai X.. (2015). Type-II Weyl semimetals. Nature 527, 495–498. 10.1038/nature15768 26607545

[B33] Son D. T. Spivak B. Z. (2013). Chiral anomaly and classical negative magnetoresistance of Weyl metals. Phys. Rev. B 88:104412 10.1103/PhysRevB.88.104412

[B34] Sun Y. Wu S. C. Yan B. H. (2015). Topological surface states and Fermi arcs of the noncentrosymmetric Weyl semimetals TaAs, TaP, NbAs, and NbP. Phys. Rev. B 92:115428 10.1103/PhysRevB.92.115428

[B35] Tian S. J. Zhang J. F. Li C. H. Ying T. P. Li S. Y. Zhang X.. (2019). Ferromagnetic van der Waals crystal VI_3._ J. Am. Chem. Soc. 141, 5326–5333. 10.1021/jacs.8b13584 30856325

[B36] Togo A. Oba F. Tanaka I. (2008). First-principles calculations of the ferroelastic transition between rutile-type and CaCl_2_-Type SiO_2_ at high pressures. Phys. Rev. B 78:134106 10.1103/PhysRevB.78.134106

[B37] Wan X. Turner A. M. Vishwanath A. Savrasov S. Y. (2011). Topological semimetal and Fermi-arc surface states in the electronic structure of pyrochlore iridates. Phys. Rev. B 83:205101 10.1103/PhysRevB.83.205101

[B38] Wang Z. J. Vergniory M. G. Kushwaha S. Hirschberger M. Chulkov E. V. Ernst A.. (2016). Time-reversal-breaking weyl fermions in magnetic heusler alloys. Phys. Rev. Lett. 117:236401. 10.1103/PhysRevLett.117.236401 27982662

[B39] Weng H. M. Fang C. Fang Z. Bernevig B. A. Dai X. (2015). Weyl semimetal phase in noncentrosymmetric transition-metal monophosphides. Phys. Rev. X 5:011029 10.1103/PhysRevX.5.011029

[B40] Wu Y. Mou D. X. Jo N. H. Sun K. W. Huang L. N. Bud'ko S. L. (2016). Observation of Fermi arcs in the type-II Weyl semimetal candidate WTe2. Phys. Rev. B 94:121113 10.1103/PhysRevB.94.121113

[B41] Xu G. Weng H. M. Wang Z. J. Dai X. Fang Z. (2011). Chern $HALL effect in HgCr_2_Se_4_. Phys. Rev. Lett. 107:186806. 10.1103/PhysRevLett.107.186806 22107665

[B42] Yang H. F. Yang L. X. Liu Z. K. Sun Y. Chen C. Peng H.. (2019). Topological Lifshitz transitions and Fermi arc manipulation in Weyl semimetal NbAs. Nat. Commu. 10:3478. 10.1038/s41467-019-11491-4 31375677 PMC6677823

[B43] You J. Y. Chen C. Zhang Z. Sheng X. L. Yang S. Y. A. Su G. (2019). Two-dimensionalWeyl half-semimetal and tunable quantum anomalous Hall effect. Phys. Rev. B 100:64408 10.1103/PhysRevB.100.064408

[B44] Yu Z. M. Yao Y. Yang S. Y. A. (2016). Predicted unusual magnetoresponse in type-II Weyl semimetals. Phys. Rev. Lett. 117:077202. 10.1103/PhysRevLett.117.077202 27563994

[B45] Zhang F. Mi W. Wang X. (2020). Spin-dependent electronic structure and magnetic anisotropy of 2D ferromagnetic Janus Cr_2_I_3_X_3_ (X = Br, Cl) monolayers. Adv. Electron. Mater. 6:1900778. 10.1002/aelm.20190077832270829

[B46] Zyuzin A. A. Tiwari R. P. (2016). Intrinsic anomalous Hall effect in type-II Weyl semimetals. JETP Lett. 103, 717–722. 10.1134/S002136401611014X

